# An Interesting Case of Acquired Renal Fanconi Syndrome

**DOI:** 10.7759/cureus.65208

**Published:** 2024-07-23

**Authors:** Subbiah Senthilnathan, Gunasekaran Nallusamy, Priyadarshini Varadaraj

**Affiliations:** 1 Internal Medicine, Saveetha Medical College and Hospitals, Saveetha Institute of Medical and Technical Sciences, Saveetha University, Chennai, IND

**Keywords:** drug-induced nephrotoxicity, heavy metals induced, ayurvedic medicine, renal tubular acidosis, renal fanconi syndrome

## Abstract

Renal Fanconi syndrome (RFS) is a proximal tubular dysfunction characterized by abnormalities in the absorption of electrolytes and substances by the proximal tubule. It can manifest as proteinuria, glycosuria, aminoaciduria, phosphaturia, and proximal renal tubular acidosis (RTA) type 2. Heavy metal-induced nephrotoxicity from heavy metals such as iron, cadmium, mercury, and lead is a common risk factor for type 2 RTA. Environmental contamination or prolonged contact with pesticides, such as lead arsenate, Bordeaux mixture, and copper sulfate, may also contribute to heavy metal exposure. A 25-year-old female patient with RFS was diagnosed, and the bicarbonate loading test showed typical features of generalized proximal tubular dysfunction and proximal RTA. A metabolic disorder in the bone was the predominant feature, and renal wasting of phosphorus and calcium was linked to osteoporosis and increased intact parathyroid hormone (PTH) levels. Due to reports of zoledronate-induced RFS, calcitriol was administered instead of bisphosphates. Drug-induced nephrotoxicity is the most probable cause of adult-onset RFS. Acute tubulointerstitial nephritis is a plausible hypothesis, and treatment was based on observations and assessments that improved clinical outcomes. Ayurvedic medicine, popular in India due to its traditional roots and cultural aversion to artificial drugs, has high levels of heavy metal content.

## Introduction

Renal Fanconi syndrome (RFS) is a proximal tubular dysfunction that is characterized by a deviation in the absorption of electrolytes and other substances by the proximal tubule. RFS can apparently manifest as proteinuria, glycosuria, aminoaciduria, phosphaturia, and proximal renal tubular acidosis (RTA) [1]. RFS involves dysfunction of the proximal tubule (PT), leading to symptoms such as proteinuria, aminoaciduria, glycosuria, and phosphaturia without affecting other nephron segments. It has been proposed that a heterozygous missense mutation causes mistargeting of the peroxisomal enzyme EHHADH in mitochondria, disrupting mitochondrial function and energy generation via fatty acid metabolism. This novel mechanism highlights the PT's reliance on mitochondrial function. Inheritance follows an autosomal dominant pattern [2]. It can be caused by a variety of factors, such as inherited disorders, including Lowe syndrome, Wilson's disease, Dent's disease, galactosemia, fructose intolerance, tyrosinemia, and mitochondrial cytopathy.

Environmental nephrotoxins, commonly encountered in the workplace, include significant industrial substances such as lead, mercury, cadmium, silica, various organic hydrocarbons, uranium, chromium, and arsenic. Lead toxicity in children has been linked to Fanconi syndrome. Mercury exposure, which can occur via inhalation, ingestion, or skin contact, leads to mitochondrial swelling and endoplasmic reticulum dilation in proximal tubular cells. Cadmium, once absorbed, accumulates in several organs, such as the bone, liver, and kidneys. Halogenated hydrocarbons have been associated with acute tubular necrosis, or Fanconi syndrome, in human and animal studies. Initially, solvent nephropathy was tied to antiglomerular basement membrane antibody-mediated glomerulonephritis and pulmonary hemorrhage, but later cases have shown various types of glomerulonephritis [3].

Heavy metal-induced nephrotoxicity from heavy metals such as cadmium, iron, mercury, and lead is a common risk factor for type 2 RTA. Such instances may be due to environmental contamination or prolonged contact with pesticides such as lead arsenate, Bordeaux mixture, and copper sulfate. Fanconi syndrome is a disorder of the proximal tubule that results in the malabsorption of various electrolytes and substances normally reabsorbed there. It can be inherited or acquired. This condition is distinct from Fanconi anemia, a rare recessive disorder characterized by pancytopenia, bone marrow hypoplasia, melanin-induced skin discoloration, and multiple congenital anomalies. In adults, Fanconi syndrome is typically acquired, while in children, it is usually genetic. Treatment depends on the specific cause and generally involves addressing the underlying issue and correcting any volumetric, nutritional, or electrolyte imbalances. Fanconi syndrome is defined by a global defect in the tubule, leading to inadequate reabsorption of solutes that the tubule would normally reabsorb [4]. The use of unregulated Ayurvedic medications with potentially high levels of heavy metals or even prolonged use of medications with acceptable levels of the same may be another potential cause of heavy metal exposure [5]. Here, we report the case of a 25-year-old female presenting with features of Fanconi syndrome.

## Case presentation

A 25-year-old female IT employee presented to the emergency department with a sudden onset of upper and lower extremity weakness associated with severe pain that started one day prior to the presentation. She gradually developed difficulty speaking and swallowing both solid and liquid diets. There was no associated fever, neck stiffness, or altered sensorium. There were no signs of increased intracranial hypertension, such as headaches, diplopia, projectile vomiting, or seizures. There was no history suggesting sensory system or cranial nerve involvement. Past medical history includes the intake of herbal medicine for the treatment of polycystic ovarian disease (PCOD) for the past three days. The general examination was unremarkable, and vitals were stable. The neurological examination showed normal higher mental function and cranial nerve function. The examination of the motor system revealed hypotonia and hyporeflexia, and the Muscle Power Assessment Scale (MRC) graded the power in the proximal and distal groups of the lower limbs at 0/5 on both sides. Power in the proximal and distal groups of the upper limbs was MRC grade 0/5 and MRC grade 1/5, respectively. The rest of the neurological exam was normal. The arterial blood gas (ABG) analysis showed a pH of 7.02, HCO_3_ of 8.4 mEq/dL, potassium of 2 mg/dL, and a calculated serum anion gap of 10, which revealed normal anion gap metabolic acidosis (NAGMA) with severe hypokalemia.

A routine urine examination revealed a pH of 6.5 and a specific gravity of 1.005. The extent of proteinuria, glucosuria, and ketonuria was 1+, 3+, and 1+, respectively. No WBC, RBC, or specific cast cells were found; the renal function test results revealed serum urea of 35 mg/dL, serum creatinine of 3.2 mg/dL, and serum uric acid of 3.3 mg/dL, suggestive of acute kidney injury. Urine spot tests revealed levels of potassium and sodium of 29.3 mEq/dL and 66 mEq/dL, respectively, suggestive of renal potassium wasting. Other laboratory indices showed urine osmolality of 204 mOsm/dL, serum osmolality of 298 mOsm/dL, serum calcium of 8 mg/dL, and serum phosphorous of 0.8 mg/dL, which were suggestive of severe hypophosphatemia and hypocalcemia. Vitamin D deficiency was identified with serum vitamin D levels of 14.2 ng/dL. Serum cortisol, perinuclear anti-neutrophil cytoplasmic antibodies (p-ANCA), C-ANCA, C3, and C4 were within the normal limit. To rule out autoimmune etiology, an anti-nuclear antibody (ANA) immunoblot test was found to be negative. Rhabdomyolysis was also ruled out by a normal serum creatine kinase (CPK) value.

Based on history and baseline investigations, NAGMA, severe hypokalemia, euglycemic glucosuria, renal potassium wasting, and phosphaturia favored the working diagnosis of type 2 RTA, probably due to herbal medicine intake. The patient was started on potassium chloride continuous infusion after central venous line placement; bicarbonate correction was started; adequate IV fluids were given; and a repeat ABG showed improvement in pH and potassium levels. The patient also showed clinical improvement in muscle power to an MRC grade of 2/5 on the day of admission.

## Discussion

The case of the 25-year-old female IT employee presenting with acute neuromuscular symptoms and metabolic derangements highlights the complexities and clinical implications of type 2 RTA, likely induced by herbal medicine for PCOD [6,7]. Type 2 RTA is characterized by impaired bicarbonate reabsorption in the PT, leading to NAGMA, as evidenced by the patient's pH and HCO_3_ levels. Severe hypokalemia and renal potassium wasting further corroborate the diagnosis, reflecting dysfunction in renal tubular handling of electrolytes [8]. In a study conducted by Clarke et al., it was shown that rarely, rickets, an outcome of mineral losses in the urine and abnormalities in vitamin D metabolism, may bring a patient to the doctor's attention. Renal wasting of phosphorus and calcium was linked to osteoporosis and increased intact PTH levels. Due to several reports of zoledronate-induced RFS, we administered calcitriol instead of bisphosphonates [9].

The clinical picture also reveals concurrent features of Fanconi syndrome, marked by glycosuria, phosphaturia, and generalized proximal tubular dysfunction [10]. This syndrome encompasses a spectrum of tubular disorders affecting solute reabsorption, commonly presenting with metabolic acidosis and electrolyte abnormalities similar to those observed in the patient. This overlap suggests a shared pathophysiological mechanism involving acute proximal tubular dysfunction, although, in this case, exacerbated by herbal medicine ingestion [11]. Herbal medicines are increasingly used for a variety of health conditions, but their potential nephrotoxic effects are often underappreciated. The lack of regulation and standardization in herbal supplements can lead to significant variability in their composition, increasing the risk of toxicity [12]. Herbal remedies, often perceived as innocuous, can exert potent nephrotoxic effects through mechanisms such as direct tubular toxicity or immune-mediated injury, as seen in cases of aristolochic acid nephropathy [13]. Vigilance in identifying and addressing such exposures is paramount to mitigating acute renal injury and optimizing patient outcomes.

Management strategies for both proximal RTA and Fanconi syndrome focus on correcting acid-base disturbances and electrolyte imbalances. Immediate interventions included potassium replacement, bicarbonate therapy for acidosis correction, and intravenous fluid administration to optimize renal function and electrolyte balance. The prompt clinical improvement in muscle power following treatment initiation underscores the critical role of early intervention in managing these complex renal disorders [12]. The differential diagnosis also considered other potential causes of proximal tubular dysfunction, such as heavy metal poisoning, autoimmune diseases, and other medications, all of which were systematically ruled out. Acute tubulointerstitial nephritis, as evidenced by the acute kidney injury with generalized proximal tubular dysfunction due to heavy metal toxicity, is a plausible hypothesis, and treatment was based on the observations and assessment that resulted in an improvement in clinical outcome [11].

This case underscores the diagnostic challenges and overlapping clinical features between proximal RTA and Fanconi syndrome, exacerbated by herbal medicine use. It highlights the importance of a systematic approach to renal tubular disorders, integrating clinical findings with biochemical investigations to guide targeted therapeutic interventions and improve patient prognosis [14,15]. Drug-induced nephrotoxicity would be the most probable cause of adult-onset RFS [6]. In our case, a certain Ayurvedic medicine taken for PCOD disorder may be the culprit of the RFS because aristolochic acid-associated nephropathy was reported to be associated with RFS [16]. The study titled "Osteomalacia associated with adult Fanconi's syndrome" found that patients with RFS did not exhibit any morphological findings. However, the renal tissue morphological examination revealed no pathology in our case as well [10]. RFS has been shown to exacerbate systemic illnesses such as Legionella pneumonia and monoclonal gammopathy [17]. Our clinical and laboratory examinations ruled out these possible scenarios.

Long-term management of patients with proximal RTA and Fanconi syndrome involves continuous monitoring of renal function, electrolyte levels, and bone health. Patient education on avoiding nephrotoxic substances, including certain herbal medicines, is crucial in preventing recurrence. Further research and awareness are needed to understand the full spectrum of nephrotoxicity associated with herbal medicines and to develop specific guidelines for their safe use.

## Conclusions

Ayurvedic medicine has been popular for a number of ailments in India, given its traditional roots and a cultural aversion to the so-called artificial drugs used in modern medicine. There is, however, a dearth of quality control and oversight in the manufacturing process of these alternative forms of medicine. Studies consistently show high levels of heavy metal content in Ayurvedic medicines. Lead, arsenic, and copper in herbal medicines have been documented to be the cause of heavy metal toxicity across multiple case series. The human body requires heavy metals in regulated doses, but an overdose of the same results in systemic illnesses, including RTA. Surveillance of these drugs in terms of their content, over-the-counter availability, and chronic consumption is pivotal.

This case underscores the importance of considering drug-induced nephrotoxicity in adult-onset RFS and highlights the need for awareness of the potential renal side effects of herbal medicines. The absence of morphological abnormalities in renal tissue aligns with previous findings in RFS cases. Clinicians should maintain a high index of suspicion for proximal tubular dysfunction when patients present with acute muscle weakness and metabolic acidosis, especially when there is a history of exposure to potential nephrotoxins. Early diagnosis and prompt treatment are crucial for improving patient outcomes, as demonstrated by the patient's rapid recovery of muscle power and stabilization of biochemical parameters. This case also emphasizes the need for careful monitoring and regulation of alternative medicine usage to prevent similar adverse effects.
